# Correction: The Lyme disease agent co-opts adiponectin receptor-mediated signaling in its arthropod vector

**DOI:** 10.7554/eLife.77794

**Published:** 2022-02-18

**Authors:** Xiaotian Tang, Yongguo Cao, Gunjan Arora, Jesse Hwang, Andaleeb Sajid, Courtney L Brown, Sameet Mehta, Alejandro Marín-López, Yu-Min Chuang, Ming-Jie Wu, Hongwei Ma, Utpal Pal, Sukanya Narasimhan, Erol Fikrig

**Keywords:** Mouse, Other

Tang X, Cao Y, Arora G, Hwang J, Sajid A, Brown CL, Mehta S, Marín-López A, Chuang Y-M, Wu M-J, Ma H, Pal U, Narasimhan S, Fikrig E. 2021. The Lyme disease agent co-opts adiponectin receptor-mediated signaling in its arthropod vector. *eLife* **10**:e72568. doi: 10.7554/eLife.72568.

Published 16, November 2021

#1

After publication, we noticed that the figure of no transfection control (Fig. 4D, left) is not the correct one we originally used. It doesn't have the same magnification compared to another figure (Fig. 4D, right) in the same panel. This is because we inadvertently pasted Fig. 4G (empty pEZT vector control, left) twice in Prism. The Fig. 4G covered the original Fig. 4D. We then moved the top Fig. 4G to its current place, and adjusted all the figures to the current size without noticing the extra one. Fig. 4D (left) and Fig. 4G (left) are both negative controls with no signal. The correction of Fig. 4D (left) doesn’t affect any text, results and conclusion in the paper.

The corrected Figure 4 (panel D left) is shown here:

**Figure 1:**
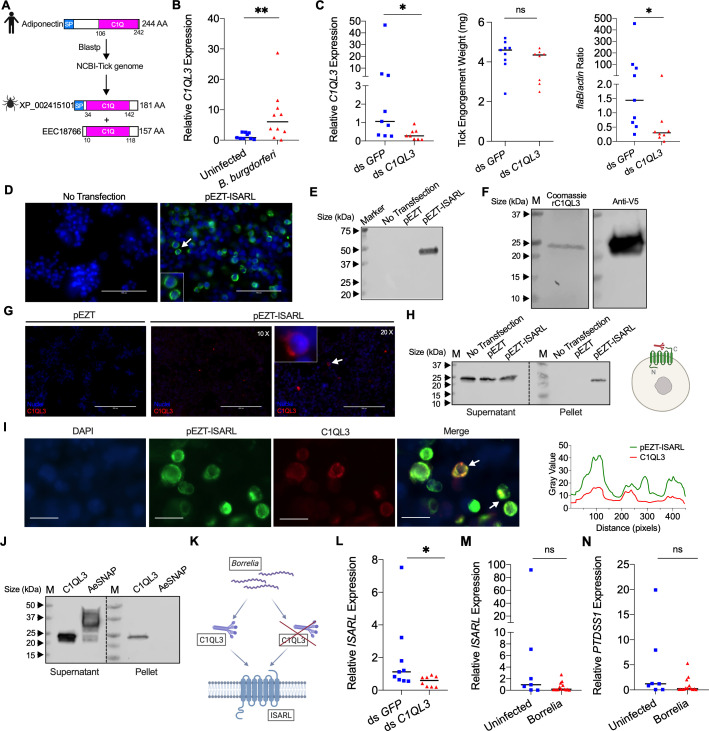


The originally published Figure (panel D left) is shown here for reference:

**Figure 2:**
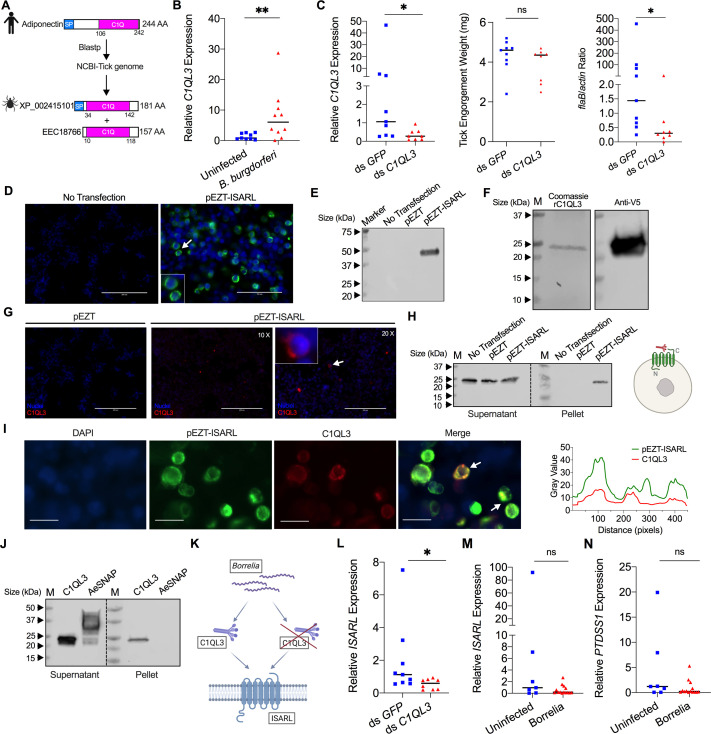


#2

In the key resources table, we omitted to change Adipo^-/-^ C57BL/6 J to Adipo**q**^-/-^ C57BL/6 J in the second round of review.

Original text:

Adipo^-/-^ C57BL/6 J

Corrected text:

Adipoq^-/-^ C57BL/6 J

The article has been corrected accordingly.

