# Supplementary figures and images for: The Lyme disease agent co-opts adiponectin receptor-mediated signaling in its arthropod vector
Source: eLife. 2021 Nov 16;10:e72568. doi: 10.7554/eLife.72568 (PMC8639152; doi:10.7554/eLife.72568)

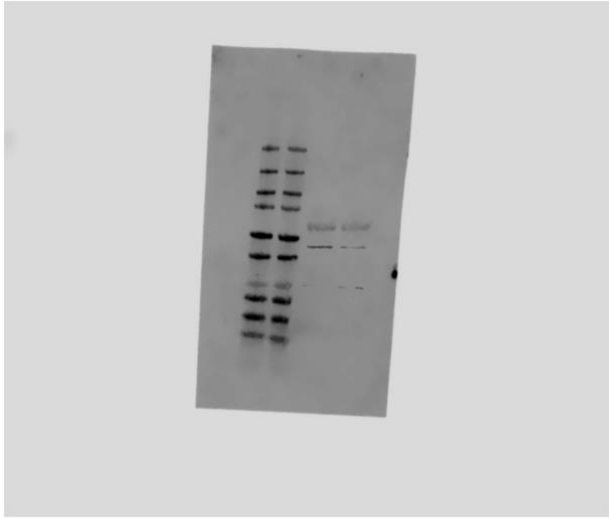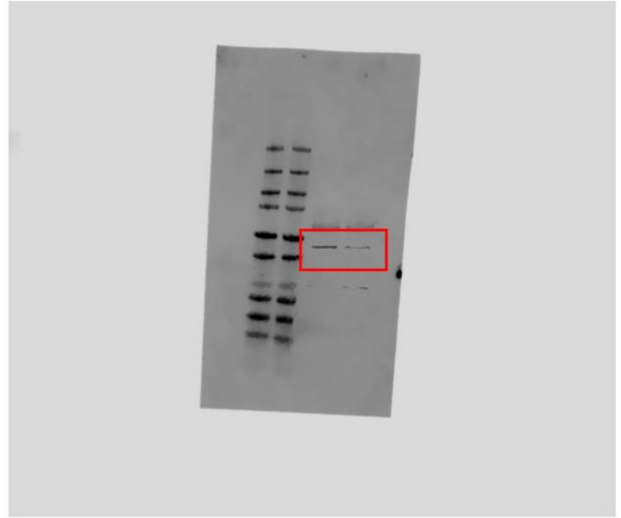

Supplement: Figure 2—source data 1. [file elife-72568-fig2-data1.pdf]

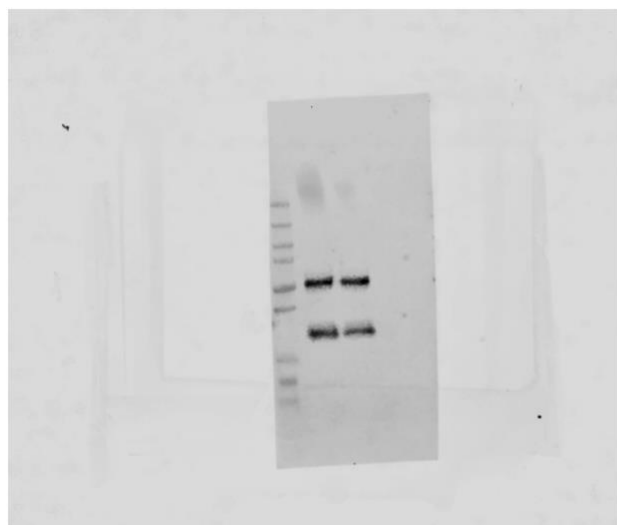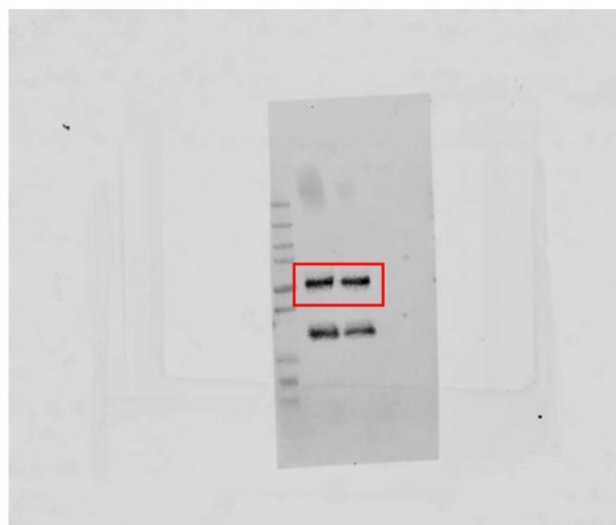

Supplement: Figure 2—source data 2. [file elife-72568-fig2-data2.pdf]

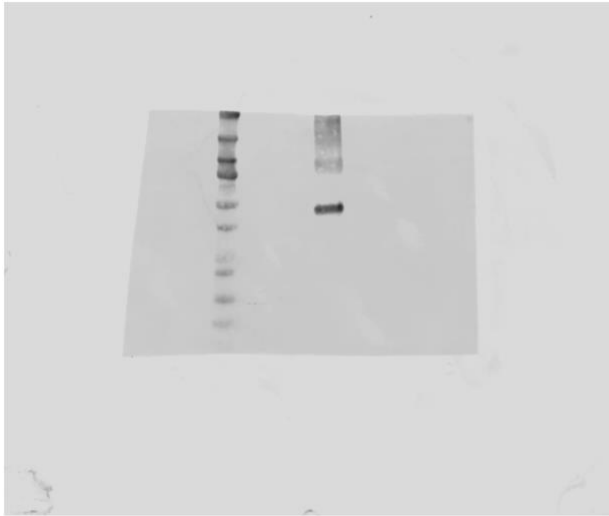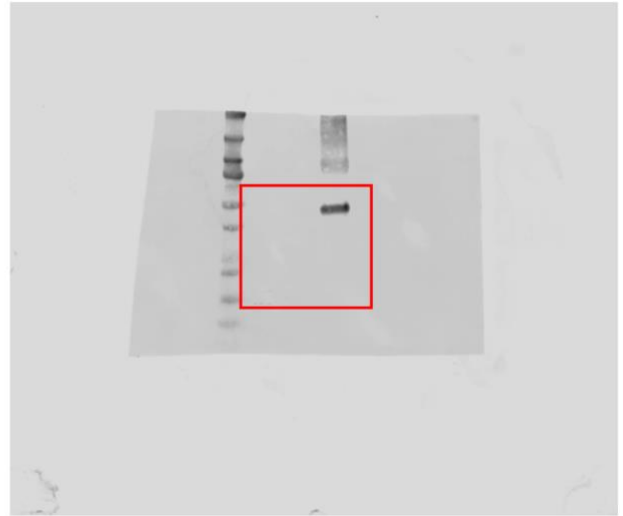

Supplement: Figure 4—source data 1. [file elife-72568-fig4-data1.pdf]

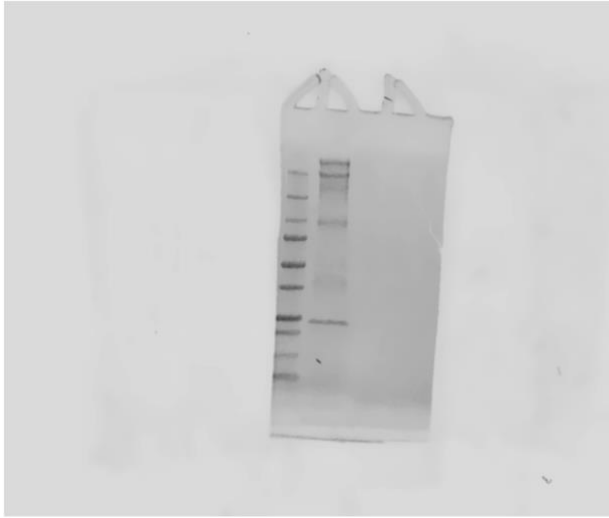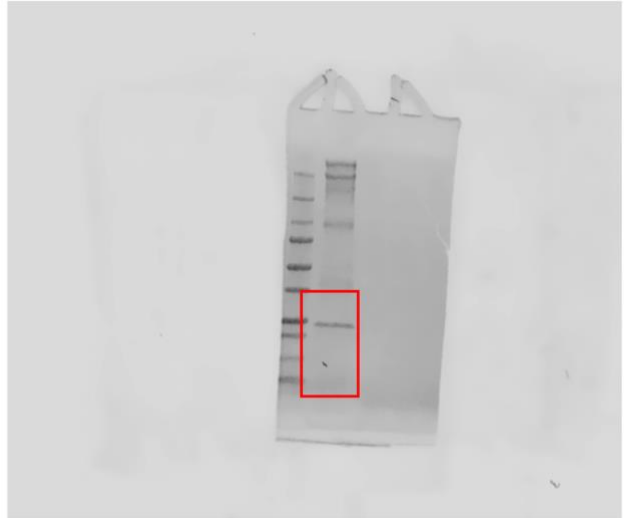

Supplement: Figure 4—source data 2. [file elife-72568-fig4-data2.pdf]

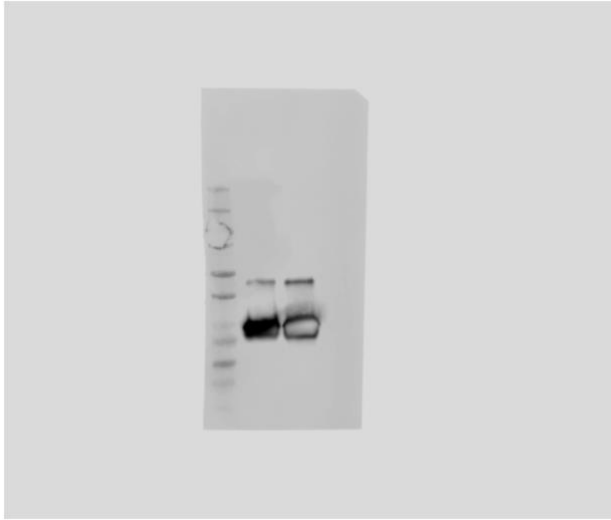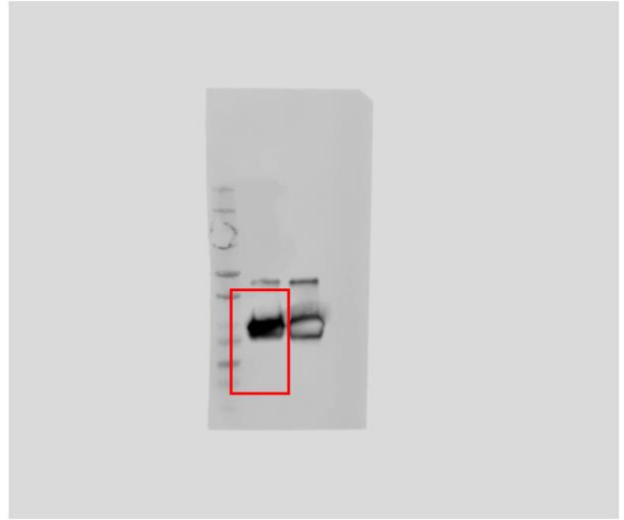

Supplement: Figure 4—source data 3. [file elife-72568-fig4-data3.pdf]

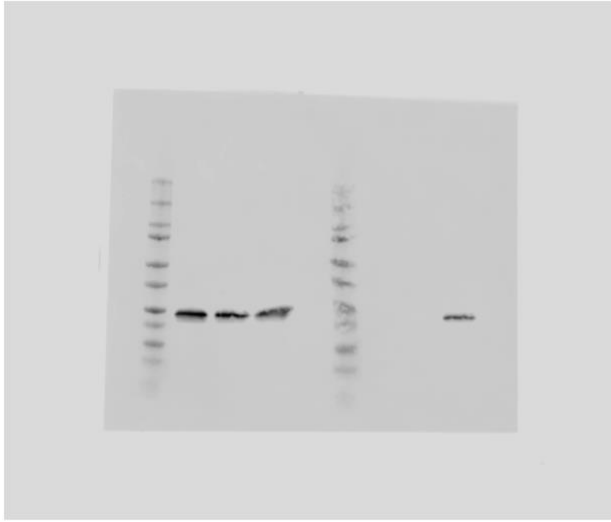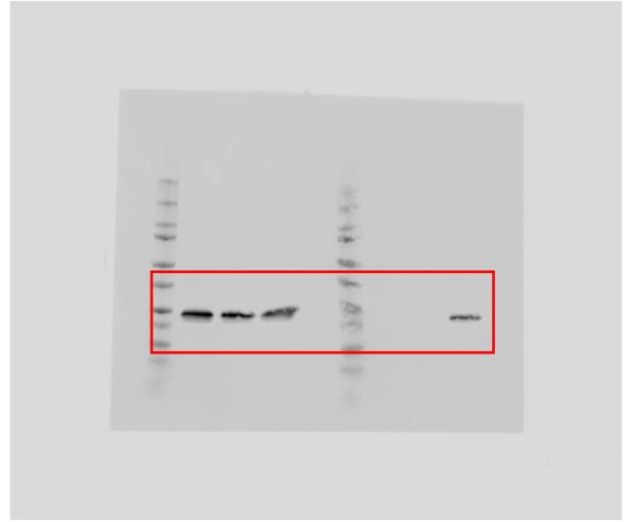

Supplement: Figure 4—source data 4. [file elife-72568-fig4-data4.pdf]

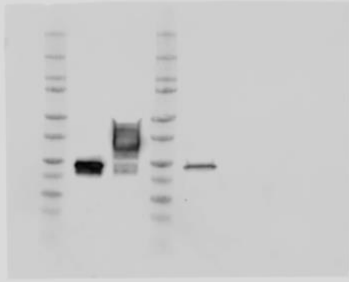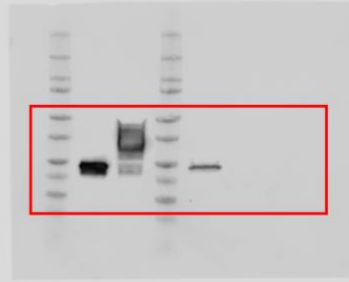

Supplement: Figure 4—source data 5. [file elife-72568-fig4-data5.pdf]
