# Supplementary material for: The Lyme disease agent co-opts adiponectin receptor-mediated signaling in its arthropod vector
Source: eLife. 2021 Nov 16;10:e72568. doi: 10.7554/eLife.72568 (PMC8639152; doi:10.7554/eLife.72568)
Supplement: Supplementary file 1. — (a) Summary of differently expressed genes of comparison between ds GFP and ds ISARL injection after 96 hr feeding on clean mice. (b) Summary of differently expressed genes of comparison between ds GFP and ds ISARL injection after 96 hr feeding on B. burgdorferi-infected mice. (c) Summary of differently expressed genes of comparison between recombinant GFP and adiponectin proteins injection after 8 hr. (d) The primers used in this study. [file elife-72568-supp1.docx]

**Supplementary file 1a. Summary of differently expressed genes of comparison between ds *GFP* and ds *ISARL* injection after 96h feeding on clean mice.**

| **Gene** | **Annotation** | **Gene Name** | **Log2FoldChange** | **P-value** |
| --- | --- | --- | --- | --- |
| ISCW003049-RA | Soluble maltase-glucoamylase, putative (Fragment) | MGA | 6.956779911 | 3.80E-06 |
| ISCW007360-RA | Gamma glutamyl transpeptidase, putative | GGT | 6.22827548 | 5.26E-07 |
| ISCW024014-RA | Gamma glutamyl transpeptidase, putative (Fragment) | GGT | 6.190152254 | 5.27E-06 |
| ISCW024661-RA | Gamma glutamyl transpeptidase, putative (Fragment) | GGT | 6.190152254 | 5.27E-06 |
| ISCW012872-RA | Gamma-glutamyltransferase, putative (Fragment) | GGT | 6.012757202 | 2.94E-11 |
| ISCW009571-RA | Uncharacterized protein (Fragment) | ISCW009571 | 3.298128524 | 2.41E-05 |
| ISCW024915-RA | REX4, RNA exonuclease 4 (S. cerevisiae) family protein (Fragment) | REX4 | -0.549991457 | 2.24E-05 |
| ISCW024422-RA | Superoxide dismutase, putative | SOD | -1.11031654 | 5.27E-05 |
| ISCW012336-RA | Superoxide dismutase [cu-zn], putative | CuZnSOD | -1.117657247 | 3.62E-05 |
| ISCW005879-RA | Uncharacterized protein | ISCW005879 | -1.608105744 | 1.35E-05 |
| ISCW012069-RA | Uncharacterized protein | ISCW012069 | -1.810228584 | 2.52E-06 |
| ISCW009622-RA | Uncharacterized protein | ISCW009622 | -2.535341888 | 1.26E-06 |
| ISCW018477-RA | Adiponectin receptor, putative (Fragment) | ISARL | -2.75905145 | 3.57E-22 |
| ISCW003837-RA | G2/mitotic-specific cyclin A, putative (Fragment) | cyclinA | -3.145555868 | 1.43E-06 |
| ISCW002566-RA | Acyl-CoA synthetase, putative (Fragment) | ACS | -3.53392918 | 5.23E-05 |
| ISCW008490-RA | Gamma glutamyl transpeptidase, putative | GGT | -5.476120094 | 5.66E-08 |
| ISCW013319-RA | Uncharacterized protein | ISCW013319 | -7.608882724 | 2.05E-07 |
| ISCW009608-RA | Glutathione S-transferase, putative | GST | -7.655953275 | 1.37E-05 |

“-” indicates downregulation of genes in the guts of ds *ISARL*-injected ticks when compared to that in control ds *GFP*-injected tick guts.

**Supplementary file 1b. Summary of differently expressed genes of comparison between ds *GFP* and ds *ISARL* injection after 96h feeding on *B. burgdorferi*-infected mice.**

| **Gene** | **Annotation** | **Gene Name** | **Log2FoldChange** | **P-value** |
| --- | --- | --- | --- | --- |
| ISCW003218-RA | FADdependent oxidoreductase domain-containing protein 2, putative | FOXRED2 | -2.879056472 | 0.000239 |
| ISCW005667-RA | Secreted protein, putative | Reeler | -2.678543731 | 0.000119 |
| ISCW003629-RA | Secreted protein, putative | ISCW003629 | -2.426582135 | 0.0001 |
| ISCW018477-RA | Adiponectin receptor, putative (Fragment) | ISARL | -1.912092255 | 1.72E-11 |
| ISCW011292-RA | Cyclic nucleotidebinding domain-containing protein | CNBD | -1.620397316 | 0.00021 |
| ISCW003135-RA | Cytochrome p450, putative | CYP | -1.427275425 | 7.35E-05 |
| ISCW001955-RA | AMP dependent CoA ligase, putative | AMP | -1.427082757 | 0.00027 |
| ISCW002185-RA | Uncharacterized protein | ISCW002185 | -1.251407193 | 3.40E-05 |
| ISCW024631-RA | 3-hydroxyacyl-CoA dehydrogenase, putative (Secreted salivary gland peptide, putative) | 3HADH | -1.089854316 | 2.66E-05 |
| ISCW013566-RA | Alpha-actinin, putative | ACTN | -1.051823169 | 4.99E-05 |
| ISCW020505-RA | Uncharacterized protein | ISCW020505 | -1.038498406 | 4.11E-05 |
| ISCW019656-RA | Secreted salivary gland peptide, putative (Fragment) | ISCW019656 | -1.000151879 | 5.89E-05 |
| ISCW016391-RA | Cytochrome P450, putative | CYP | -0.983747879 | 0.000348 |
| ISCW006151-RA | Transport protein, putative (Fragment) | ISCW006151 | -0.869973215 | 1.75E-05 |
| ISCW021203-RA | Uncharacterized protein | ISCW021203 | -0.826491344 | 9.19E-05 |
| ISCW024161-RA | Uncharacterized protein | ISCW024161 | -0.753881171 | 4.99E-05 |
| ISCW018609-RA | Hemomucin, putative (Fragment) | Hmu | -0.730567529 | 0.000361 |
| ISCW017282-RA | Oxodicarboxylate carrier protein, putative | ODC | -0.701243498 | 4.69E-05 |
| ISCW009471-RA | Phospholipase B-like (Fragment) | PLB | -0.694463465 | 8.01E-05 |
| ISCW006150-RA | Sugar transporter, putative | ISCW006150 | -0.666272429 | 6.70E-05 |
| ISCW012299-RA | Adenylosuccinate synthetase (Fragment) | ADSS | -0.649923076 | 0.00038 |
| ISCW022913-RA | cAMP and cAMP-inhibited cGMP 3,5-cyclic phosphodiesterase, putative | PDE | -0.612276289 | 3.11E-06 |
| ISCW000885-RA | Alpha-1,4 glucan phosphorylase | GP | -0.588614232 | 0.000254 |
| ISCW019117-RA | Pyridoxine kinase, putative (Fragment) | PDXK | -0.535676385 | 0.000478 |
| ISCW018543-RA | GMP synthase, putative (Fragment) | GMPS | -0.53194689 | 0.000465 |
| ISCW004028-RA | Phosphatidylserine synthase I, putative | PTDSS1 | -0.529598437 | 3.12E-05 |
| ISCW012521-RA | Uncharacterized protein | ISCW012521 | -0.517853294 | 2.92E-05 |
| ISCW009548-RA | Uncharacterized protein | ISCW009548 | -0.504914087 | 0.000199 |
| ISCW015732-RA | RNA polymerase II transcription elongation factor, putative | Elongin-C | -0.48942716 | 4.89E-05 |
| ISCW022144-RA | N-CAM Ig domain-containing protein, putative | Ncam | -0.437192969 | 0.000289 |
| ISCW009549-RA | Selenoprotein P precursor, putative | Selenop | -0.436460074 | 0.000423 |
| ISCW013692-RA | V-type proton ATPase subunit G | V-ATPase | -0.414399839 | 0.000311 |
| ISCW010157-RA | Receptor expression-enhancing protein | REEP | -0.405156446 | 0.000104 |
| ISCW023312-RA | Sidoreflexin | SFXN | -0.3999554 | 0.000229 |
| ISCW006433-RA | AP complex subunit sigma (Fragment) | APS | -0.395796427 | 0.000508 |

“-” indicates downregulation of genes in the guts of ds *ISARL*-injected ticks when compared to that in control ds *GFP*-injected tick guts.

**Supplementary file 1c. Summary of differently expressed genes of comparison between recombinant *GFP* and adiponectin proteins injection after 8h.**

| **Gene** | **Annotation** | **Log2FoldChange** | ***P*-value** |
| --- | --- | --- | --- |
| ISCW004553-RA | Cuticle protein, putative | 18.51302808 | 2.05E-12 |
| ISCW002039-RA | Cuticle protein, putative | 17.49563794 | 2.29E-07 |
| ISCW001782-RA | Uncharacterized protein | 17.27754175 | 7.09E-10 |
| ISCW013798-RA | Cuticle protein, putative | 17.20315541 | 7.96E-05 |
| ISCW015495-RA | Secreted protein, putative (Fragment) | 17.15463748 | 0.00016026 |
| ISCW005191-RA | Secreted glycine rich protein, putative (Fragment) | 17.11093994 | 2.95E-08 |
| ISCW003789-RA | Uncharacterized protein | 16.81877212 | 2.70E-06 |
| ISCW016297-RA | Uncharacterized protein | 16.74612219 | 1.13E-06 |
| ISCW008562-RA | Cuticle protein, putative | 15.3237752 | 1.02E-06 |
| ISCW002925-RA | Uncharacterized protein | 2.857394251 | 1.82E-05 |
| ISCW024478-RA | Secreted cysteine rich protein, putative (Fragment) | 2.851254132 | 1.38E-05 |
| ISCW021558-RA | Secreted salivary gland peptide, putative | 2.689268024 | 3.51E-05 |
| ISCW021555-RA | Uncharacterized protein | 2.656702948 | 4.30E-05 |
| ISCW023547-RA | Secreted salivary gland peptide, putative | 2.589439049 | 0.0001213 |
| ISCW023623-RA | Serpin-4 precursor, putative (Serpin-4, putative) | 2.173997552 | 5.42E-05 |
| ISCW008209-RA | Hebreain, putative | 2.158930697 | 1.20E-06 |
| ISCW024733-RA | Beat protein, putative (Fragment) | 2.105356324 | 1.12E-05 |
| ISCW024387-RA | Serpin-2 precursor, putative (Fragment) | 2.00955178 | 5.56E-05 |
| ISCW002113-RA | Antimicrobial peptide microplusin | 1.940836315 | 0.00014064 |
| ISCW011893-RA | ANK_REP_REGION domain-containing protein | 1.808330643 | 1.28E-08 |
| ISCW015113-RA | Uncharacterized protein | 1.783621042 | 6.95E-05 |
| ISCW012685-RA | Myosin light chain 1, putative | 1.61605594 | 9.51E-05 |
| ISCW005837-RA | Uncharacterized protein | 1.582477028 | 3.43E-05 |
| ISCW024686-RA | Ixoderin, putative (Fragment) | 1.545371762 | 3.67E-06 |
| ISCW014652-RA | Serpin-8 precursor, putative | 1.431124914 | 1.89E-06 |
| ISCW009063-RA | Tropomyosin, putative | 1.358977546 | 0.00014404 |
| ISCW023442-RA | Uncharacterized protein | 1.288696108 | 0.00015793 |
| ISCW023441-RA | Troponin, putative (Fragment) | 1.2703018 | 0.00010777 |
| ISCW016762-RA | LIM domain-containing protein, putative | 1.268776381 | 6.91E-06 |
| ISCW002637-RA | Reductase, putative | 1.195839092 | 5.26E-05 |
| ISCW017459-RA | Glucose-6-phosphatase | -1.182920138 | 0.00012321 |
| ISCW015064-RA | Cytochrome P450, putative | -1.276584666 | 5.75E-06 |
| ISCW015956-RA | Serine/threonine protein kinase, putative | -1.438675023 | 1.25E-05 |
| ISCW016390-RA | Cytochrome P450, putative | -1.643963635 | 3.32E-06 |
| ISCW016391-RA | Cytochrome P450, putative | -1.678990788 | 6.27E-05 |
| ISCW006560-RA | Cytochrome P450, putative | -2.209859052 | 1.42E-06 |
| ISCW024348-RA | Salivary HBP family protein, putative (Fragment) | -3.822772331 | 2.76E-05 |
| ISCW008563-RA | Cuticle protein, putative | -14.56782308 | 1.01E-06 |
| ISCW005940-RA | Elongation of very long chain fatty acids protein | -15.77527062 | 7.81E-08 |
| ISCW012372-RA | Cysteine rich secreted peptide, putative | -17.69968417 | 3.20E-09 |

“-” indicates downregulation of genes in the guts of adiponectin-injected ticks when compared to that in control GFP-injected tick guts.

**Supplementary file 1d. The primers used in this study.**

| **Gene name** | **Primer sequence** |
| --- | --- |
| Tick *actin* | F: GGCGACGTAGCAG R: GGTATCGTGCTCGACTC |
| Mouse *β-actin* | F: AGCGGGAAATCGTGCGTG  R: CAGGGTACATGGTGGTGCC |
| *Borrelia* *flaB* | F: TTCAATCAGGTAACGGCACA  R: GACGCRRGAGACCCTGAAAG |
| ds *GFP* | F: *TAATACGACTCACTATAGGGAGA*GCGACGTAAACGGCCACAAGTT  R: *TAATACGACTCACTATAGGGAGA*CGGGTCTTGTAGTTGCCGTC |
| ds *ISARL* | F: *TAATACGACTCACTATAGGGAGA*GACGATGACGAGGATGAGC R: *TAATACGACTCACTATAGGGAGA*CGTGTGGAAGGTGAAGGAC |
| *ISARL* qPCR | F: TGCAGGACAACGACTACCTG  R: ACCAGATGTTCCCGGTCTC |
| *ISARL*_pEZT_Dlux | F: AGGCGTTCAGTCTAGAATGGAGGTCCGCGAGCGACG  R: AGACCGGCGGCCGCTCAAGCGTAATCTGGAACATCGTATGGGTAGTCGAATGGTGGACCCTCCT |
| 3HADH qPCR | F: GACCCCCTGATTGTTATTCG  R: GCCATCCACTTCTTTCTTGA |
| ADSS qPCR | F: CACCGAGCAGAAGAACGAG  R: GAGTAGCGGAGAACCACCAG |
| GMPS qPCR | F: CACCTTCATCACCCAGGACT  R: TCTCGCTCACCATCTTCTTG |
| FOXRED2 qPCR | F: CCATCAACAACGACCTCTT  R: GGCGTCCTAACTATCTTCAGT |
| Reeler qPCR | F: CCTGGAGGAACCTGAAGAAG  R: AATGGCGTGGACGAAGTAAT |
| AMP qPCR | F: TACCACAACAAGCCACAAGC  R: CGATGTAGAACTGCCCACTC |
| ODC qPCR | F: GTCTCACGGAGGCTGTCTTC  R: TACGTGCTACGGCAAAGGTA |
| PDE qPCR | F: GACGGTGCGAAAGAACTACC  R: TTGAATGCTCCTGTGGAATG |
| GP qPCR | F: GTGGAGATGCGAGAGGAGAT  R: GTAGTCCCAGGCGTTGTAGC |
| PTDSS1 qPCR | F: ATGGCTTCGGCATCTTCTT  R: TCGTGGTCTGAATGTCCTTG |
| Ncam qPCR | F: GCTGCGGGAGAACTATGTG  R: CTTGTTGAGGTGTTGCTGCT |
| VATPase qPCR | F: ATGGCTAGTCAAAGCCAAGG  R: CATCGGCGACTTTTTCAGAC |
| ACTN qPCR | F: ACCGCTACACCCAGTACACC  R: TCTCGACCTCGTTGATGTTG |
| HMU qPCR | F: TCGACGCTTACTACGGTGTC  R: GTCGTCCAGAAAGAGGATGC |
| PLB qPCR | F: GAATTTTCTCTGGCGACGAC  R: AAGAGTTGCCGTTCCCTGT |
| PDXK qPCR | F: CTGAAAGAGGACAACCCTTCA  R: GCTCCCTGTAGATGCTCACC |
| Selenop qPCR | F: CAGTGCAAGAAACTCCACCA  R: AAAGTCTGGACGCCTTCGTA |
| SFXN qPCR | F: TCTCTGCGGTCTTCTGCTC  R: CGAACCACCTCCTGAATCTC |
| ds *3HADH* | F: *TAATACGACTCACTATAGGGAGA*GTTGCACTCTTTGACGTGGA  R: *TAATACGACTCACTATAGGGAGA*AGTGGGACGTAGTATGGTGGA |
| ds *PTDSS1* | F: *TAATACGACTCACTATAGGGAGA*CTAGTCAAAAGCCCGACCAC  R: *TAATACGACTCACTATAGGGAGA*TGACCGCATACTCCTTCTCA |
| ds *ADSS* | F: *TAATACGACTCACTATAGGGAGA*CAGTGGTGAACAGCGTGAA  R: *TAATACGACTCACTATAGGGAGA*TTGGTGGAAGTCAAAAACGA |
| ds *GMPS* | F: *TAATACGACTCACTATAGGGAGA*AAGGACTTCCACAAGGACGA  R: *TAATACGACTCACTATAGGGAGA*ACACGTACACCACCCTGTTG |
| ds *NCAM* | F: *TAATACGACTCACTATAGGGAGA*ACTTTGGAGGTGCTGGACA  R: *TAATACGACTCACTATAGGGAGA*AGAGAGTGGCAGACGGAGAC |
| ds *ACTN* | F: *TAATACGACTCACTATAGGGAGA*ATCTGCTGGACTACGGGAAG  R: *TAATACGACTCACTATAGGGAGA*GGGTGTTGAAGTTGGTCTCC |
| ds *SFXN* | F: *TAATACGACTCACTATAGGGAGA*TTGGGACCAGAGCACCTACT  R: *TAATACGACTCACTATAGGGAGA*GAAGCGTCCTACCAGAGGAG |
| ds *V-ATPase* | F: *TAATACGACTCACTATAGGGAGA*GAAGCAGGCAAAGGATGAAG  R: *TAATACGACTCACTATAGGGAGA*AACGTCAGGAGCTGCTCAAT |
| ds *PSD* | F: *TAATACGACTCACTATAGGGAGA*GACTACCACCGCTTCCACTC  R: *TAATACGACTCACTATAGGGAGA*CCTCGAAGATGAGCACCAC |
| PSD qPCR | F: GAAGGGCATCACCTACTCC  R: CTTCTGCTGGTACTCCTCCTC |
| G6P1 qPCR | F: AGCCTGTCCCGAATCTACA  R: CGTTGTCCGTGTCCATCTT |
| G6P2 qPCR | F: TCCATCTATTTCGGGCTGAT  R: GTTCACGTAGGTCGGGTCAT |
| PEPCK1 qPCR | F: CAACACCATTTTCACCAACG  R: AGTTTGCCTCCCTTTTCCA |
| PEPCK2 qPCR | F: TTCCACTGCCCAAGTATCG  R: GCTCCGTGCTGATGAATGT |
| PEPCK3 qPCR | F: GAGCACAAAGGCAAGGTGA  R: TTCCCAGACTCAGCCAATG |
| ds *G6P1* | F: *TAATACGACTCACTATAGGGAGA*GCCAGTGCTATGTCCACCT  R: *TAATACGACTCACTATAGGGAGA*GAGACGCCCCGATAAAGAC |
| ds *G6P2* | F: *TAATACGACTCACTATAGGGAGA*AGCACCGACCCTTCTGGTA  R: *TAATACGACTCACTATAGGGAGA*GATGACCCCACTGACTACGG |
| ds *C1QL3* | F: *TAATACGACTCACTATAGGGAGA*GAACATGCAGGCAGAAATCA R: *TAATACGACTCACTATAGGGAGA*ACGAGAAAGCCCGAGAAAG |
| C1QL3 qPCR | F: ACGAGAGCCATCACCTCCT  R: TCCCCTTTCTGCGAATAAGA |
| C1QL3_pMT | F: CTCGCTCGGGAGATCTATGCAGACCTGGGTTGTTCTTG  R: GCCCTCTAGACTCGAGTACCGTCCCCTTTCTGCGAAT |

The underlines indicate restriction enzymes sites. The italicized letters indicate T7 promoter sequence.
